# A Perspective on the Role of microRNA-128 Regulation in Mental and Behavioral Disorders

**DOI:** 10.3389/fncel.2015.00465

**Published:** 2015-12-14

**Authors:** Ai-Sze Ching, Azlina Ahmad-Annuar

**Affiliations:** Department of Biomedical Science, Faculty of Medicine, University of MalayaKuala Lumpur, Malaysia

**Keywords:** microRNAs, gene expression, neuropsychiatric disorders, anxiety, fear, movement disorders

## Abstract

MiRNAs are short, non-coding RNA molecules that regulate gene expression post-transcriptionally. Over the past decade, misregulated miRNA pathways have been associated with various diseases such as cancer, neurodegenerative diseases, and neurodevelopmental disorders. In this article, we aim to discuss the role played by *miR-128* in neuropsychiatric disorders, and highlight potential target genes from an *in silico* analysis of predicted *miR-128* targets. We also discuss the differences of target gene determination based on a bioinformatics or empirical approach. Using data from TargetScan and published reports, we narrowed the *miR-128* target gene list to those that are known to be associated with neuropsychiatric disorders, and found that these genes can be classified into 29 gene clusters and are mostly enriched in cancer and MAPK signaling pathways. We also highlight some recent studies on several of the *miR-128* targets which should be investigated further as potential candidate genes for therapeutic interventions.

## Introduction

Mature microRNAs (miRNAs) are endogenous, small (approximately 22 nucleotides long), non-coding RNA molecules which regulate their target genes post-transcriptionally by targeting the 3′ untranslated region (UTR), 5′UTR and coding regions of target messenger RNAs (mRNAs) thus causing translational repression or mRNA degradation (Lewis et al., 2005; Bartel, 2009; Lee et al., 2009; Schmiedel et al., 2015).

MicroRNA-128 (*miR-128*) is an intronic miRNA and the mature *miR-128* form is encoded by the two isoforms; *miR-128-1* and *miR-128-2* (Megraw et al., 2010). The *pri-miR-128-1* gene resides within the R3H domain containing protein 1 gene (*R3HDM1*) and *pri-miR-128-2* lies within the cAMP-regulated phosphoprotein, 21 kDa gene (*ARPP21*, also known as regulator of calmodulin signaling, *RCS*). This organization is conserved in human, rat and mouse genomes.

Early studies on *miR-128* pointed to its tumor suppressive activity. Loss of *MIR-128* was reported in human lung cancers – due to a deletion in chromosome 3p which included the *MIR-128-2* and *ARPP21* locus (Weiss et al., 2008) and in breast cancer (Qian et al., 2012).

Apart from its anti-cancer activity, *miR-128* is of particular interest to neuroscientists as it is a brain-enriched miRNA which is highly expressed in the cortex and cerebellum, with distinct patterns of expression in developing brains and maturing cortical neurons (Krichevsky et al., 2003; Kim et al., 2004; Smirnova et al., 2005). Overexpression of *miR-128* promotes neuronal differentiation in P19 cells and in primary embryonic neural stem cells through the suppression of non-sense-mediated decay (Bruno et al., 2011; Karam and Wilkinson, 2012). *MiR-128*- transduced human induced pluripotent stem cells shows similar characteristics as mature neurons and enhances the expression of beta-tubulin and other neuronal markers (Zare et al., 2015).

Recent studies have begun to relate *MIR-128* and *ARPP21* with neuropsychiatric disorders such as fear response, anxiety, intellectual disability and in movement disorders (Lin et al., 2011; Davis et al., 2012; Marangi et al., 2013;Tan et al., 2013). Tan et al. (2013) observed early onset fatal epilepsy and increased neuronal activity in mice with *miR-128* deficiency. In this study, dopamine-1 receptor neurons with suppressed *miR-128* expression showed enhanced dendritic excitability and increased dendritic spine formation. Recently, a family with a deletion of the *MIR-128-2* and *ARPP21* locus (chromosome 3p22.3p22.2) was reported to present with intellectual disability and epileptic episodes (Marangi et al., 2013). Several family members also had two other chromosomal abnormalities (3p24.3 deletion and 6p22.31 duplication), and suffered from febrile seizures during early childhood and decreased muscle tone. However, no direct link to *ARPP21* or *MIR-128* was discussed in the paper. It is interesting to note that a deletion in the same locus can also give rise to lung cancer, mentioned above (Weiss et al., 2008). The actual phenotype that manifests may be subject to environmental factors or other uncharacterised genetic factors.

Further evidence of the key role of *MIR-128* in cognitive function comes from studies where mice were trained on a fear-conditioning paradigm, and the level of *miR-128* expression was observed to increase upon learning the associated tasks (Lin et al., 2011). Interestingly, knockout *Arpp21/Rcs* mice showed anxiety-like behavior and had decreased motivation in food-rewarded tasks (Davis et al., 2012). However, in these papers there is no data to directly link *miR-128* to these phenotypes and it may be that the abnormalities seen could involve other pathways, perhaps through *Arpp21/Rcs* direct interaction with calmodulin.

Several lines of evidence point to *miR-128* and *Arpp21* being susceptible to pharmacological modulation in relation to schizophrenia and depression. Rats administered with the antipsychotic drug, haloperidol, exhibited an increase in the expression of *miR-128* in the prefrontal cortex, but no differences were seen in human schizophrenic patients when compared to controls (Perkins et al., 2007). *MIR-128-1* overexpression has been also reported in the dorsolateral prefrontal cortices of schizophrenic patients (Beveridge et al., 2010) and patients with major depression treated with selective serotonin reuptake inhibitor antidepressant treatments, showed an upregulation of *MIR-128* in their blood (Bocchio-Chiavetto et al., 2013). However, these studies do not conclusively show that the *MIR-128* upregulation was due to the psychiatric disorders alone, or whether the elevation was in part due to the drug treatment.

Given that there is mounting evidence that *MIR-128* is important in neuropsychiatric/ neurological disorders, and the recent Tan et al. (2013) report listing 1061 target genes of *miR-128*, we aimed to pull together results from numerous reports to determine what were the most likely candidate genes involved in this pathological process. We compared the target gene dataset from Tan et al. (2013) with datasets obtained through TargetScan analysis and searched the literature for reported target genes, to enable further exploration into their potential neuropsychiatric role. Using this approach, we narrowed down the list of potential target genes to 108. These genes can be classified into several functional clusters and share biological pathways that are relevant as possible therapeutic targets for neuropsychiatric disorders.

## *In Silico* Analysis For *miR-128* Target Genes

**FIGURE 1:**
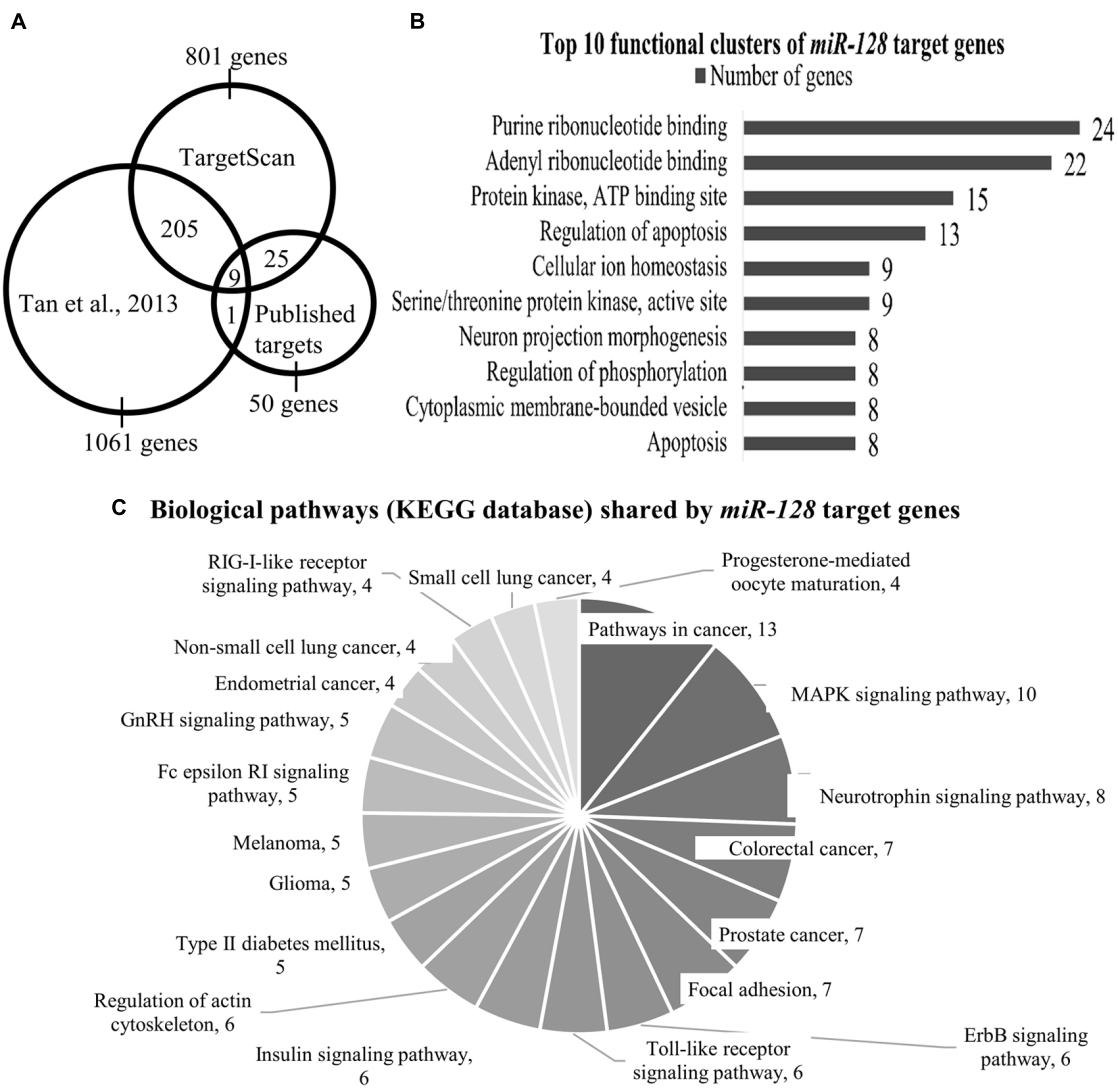
**(A)** The Venn diagram shows the number of *miR-128* target genes as identified by Tan et al. (2013) TargetScan and published targets. Total number of genes that are shared between Tan et al. (2013) and TargetScan is 214 genes; 34 genes are shared by TargetScan and our list of published *miR-128* targets. **(B)** Top 10 functional clusters of *miR-128* target genes as classified by DAVID bioinformatics tool. Using the mouse genome as the background, the threshold was set at the following criteria: classification stringency = highest; maximum EASE score = 0.05. EASE score is a modified Fisher Exact p value which range from 0 to 1, with 0 shows perfect enrichment. **(C)** Biological pathways (KEGG database) shared by *miR-128* target genes as annotated by the KEGG pathway database. Using the mouse genome as background, the threshold was set at the following criteria: minimum number of genes for the corresponding pathway = 2; maximum EASE score = 0.05.

We performed a TargetScan analysis to determine the predicted target genes of *miR-128*. TargetScan (www.targetscan.org) is an online miRNA target site prediction database which selects possible target genes based on identifying the conserved 7-8mer miRNA sequences on the 3′UTR of these genes that are complementary with the miRNA seed region (Lewis et al., 2005). This analysis identified 801 genes as potential *miR-128* targets (TargetScan release 6.2, named as Data TS, **Figure 1A**).

We then used data from Tan et al. (2013) where a HITS-CLIP (high-throughput sequencing of RNA isolated by crosslinking immunoprecipitation) approach identified 1061 genes (Dataset T). This dataset was specific to genes which were in an RNA-induced silencing complex (RISC) with *miR-128* in adult *Camk2a*-neurons. Tan et al. (2013) further defined their dataset by comparing the genes found in the RISC complex with genes that were found to be upregulated in *miR-128* knockout D1 neurons, resulting in a smaller pool of 154 genes. The majority of the genes within this smaller dataset were enriched in the ERK pathway and other pathways relevant to neuronal function like ion transport, voltage-gated ion channel activity and *G*-protein signaling.

Next, we compared the two datasets from TargetScan (801 genes) and Tan et al. (2013; 1061 genes) which indicated 214 genes were commonly shared (Data TS & T, **Figure 1A**, Supplementary Table S1).

In addition, we performed a literature review for genes that had been empirically identified as direct targets of *miR-128*. From this, 50 genes (Dataset P) were identified (source: PubMed, Scopus May 2015; Supplementary Table S2). Out of these 50 genes, 9 genes (*Arpp21*, *Bmi1*, *Csf1*, *Irs1*, *Casc3*, *Mapk14*, *Rxra*, *Snap25*, and *Sp1*) were also shared by Dataset TS & T (**Figure 1A**), meaning that these nine genes were identified by Tan et al. (2013) TargetScan and are confirmed targets of *miR-128* (**Table 1**).

**Table 1:** Neuropsychiatric association of several *miR-128* target genes from the combined datasets TS, T, and P.

Gene	Function/association with neuropsychiatric phenotypes
Bmi1 polycomb ring finger oncogene (*Bmi1*)	Promotes cell proliferation and regulates endogenous antioxidant defense pathway (Leung et al., 2004; Zencak et al., 2005).
	*Bmi1*-knockout mice exhibit epileptic-like seizures and progressive ataxia (Cao et al., 2012).
Colony stimulating factor 1 (*Csf1*)	Mediates growth and differentiation of macrophage. Target of p53 tumor suppressor gene (Azzam et al., 2013).
	Plasma *Csf1* level increased in Alzheimer’s disease patients (Laske et al., 2010).
Insulin receptor substrate 1 (*Irs1*)	Regulates insulin signaling (Zheng and Quirion, 2004).
	Downregulated in hippocampus of an Alzheimer’s disease model (Han et al., 2012).
Retinoid × receptor, alpha (*Rxra*)	Regulates cholesterol metabolism (Gentili et al., 2005).
	The rs3132293 variant is associated with a higher risk of Alzheimer’s disease (Kolsch et al., 2009).
	High deletion rate of *RXRA* in schizophrenic patients (Lee et al., 2010).
Synaptosomal associated protein, 25 kDa (*Snap25*)	Synaptosome-associated protein (Oyler et al., 1989). Involved in docking and fusion of synaptic vesicles (Sollner et al., 1993; Xu et al., 2013).
	Association with attention-deficit/ hyperactivity disorder (Faraone et al., 2005).
	Downregulated in hippocampi of schizophrenic patients (Thompson et al., 2003).
Sp1 transcription factor (*Sp1*)	A transcription factor (Dynan and Tjian, 1983). Expressed mainly in glia (Mao et al., 2009, 2014).
	Upregulates the expression of Akt-induced vascular endothelial growth factor (Pore et al., 2004).
	Regulates the expression of Huntingtin (*HTT*) gene (Wang et al., 2012).


We noticed that some of the published *miR-128* target genes were not identified by Tan’s study, such as Bcl-2 associated X protein (*Bax*; Adlakha and Saini, 2011), cAMP responsive element binding protein 1 (*Creb1*; Lin et al., 2011), Doublecortin (*Dcx*; Evangelisti et al., 2009) (*Egfr*; Weiss et al., 2008; Papagiannakopoulos et al., 2012), Neurofibromin 1 (*Nf1*; Paschou and Doxakis, 2012), Reelin (*Reln*; Evangelisti et al., 2009; Lin et al., 2011), and Wingless-related MMTV integration site 3a (*Wnt3a*; Wu et al., 2014). This may indicate several issues when determining target genes using different approaches. In particular, the target genes in Tan et al. (2013) were determined using the HITS-CLIP method with adult *Camk2a*-neurons and this method may have limited target gene identification to only genes that were expressed in those cells at that particular time. The rationale behind using these cells is justified in the Tan et al. (2013) study as they were most interested in genes that were affected by the *miR-128* deletion in dopamine responsive neurons. However, this does impose a limitation to their target gene list. TargetScan and other miRNA database prediction software are likely to produce slightly different lists as these analyses are based on bioinformatics prediction software, irrespective of the tissue expression.

Some of the genes missing from the Tan et al. (2013) dataset included genes that are known to be expressed in the brain at the time points used in the Tan et al. (2013) study. For example, *Dcx* and *Reln* are known *miR-128* targets and they are expressed in adult mouse forebrains (Evangelisti et al., 2009) including the striatum (Gates et al., 2006; Kang et al., 2011). Furthermore, knockout *Dcx* and *Reln* mice also exhibit anxiety and epileptic phenotypes, which are similar to the *miR-128* knockout mice in the Tan et al. (2013) study. It is unclear why these genes were not identified. It may be that the level of expression of these particular genes was too low to be detected or the *miR-128* regulation is specific to certain time points not included in the Tan et al. (2013) study.

When we grouped together the dataset shared exclusively between TS & T (205 genes) and included the dataset of published target genes (Dataset P, 50 genes), the number of candidate genes was 255 (Data TS and T & P). We then narrowed down the list by screening for associations with mental and behavioral disorders (as outlined by the International Statistical Classification of Diseases and Related Health Problems 10th Revision, ICD-10 version: 2010), first through the Genecard database (http://www.genecards.org/; Stelzer et al., 2011, an integrated human genes compendium), where 95 genes showed an association. The rest of the genes were subjected to systemic review on PubMed. From both of these two approaches, 109 genes out of the 255 genes (42.7%) were found to have neuropsychiatric associations (Data NP; Supplementary Figure S1 and Table S3). We then applied this list through the Database of Annotation, Visualization, and Integrated Discovery (DAVID) bioinformatics tool (version 6.7; Huang et al., 2009) to determine whether any functional clusters were enriched.

Analysis by DAVID yielded 29 functional clusters (under highest classification stringency), the Top 10 of which are shown in **Figure 1B** (the entire list can be found in Supplementary Table S4). These functional clusters indicated involvement in nucleotide binding, protein kinase activity, apoptosis, neuronal development, ion homeostasis, and maintaining cytoplasmic membrane-bound vesicles (**Figure 1B**).

The KEGG annotation tool in DAVID further identified 20 shared biological pathways (**Figure 1C**; Supplementary Table S5). We found several relevant neuropsychiatric pathways, such as the MAPK signaling pathway, neurotrophin signaling and regulation of the actin cytoskeleton which is related to neuronal morphology. The involvement of the MAPK pathway has been demonstrated by Tan et al. (2013), where they found that many genes in their target dataset were associated with the ERK pathway, and *Erk2* elevation was seen in the *miR-128* knockout mice. Cancer-associated pathways accounted for a large fraction of the genes identified (for example, in colorectal, lung, and prostate cancers), (**Figure 1C**). The role of *miR-128* and its target genes in cancer has been discussed in an excellent review (Li et al., 2013) and we will not discuss this aspect further.

## Candidate *miR-128* Target Genes With Relevant Associations To Neuropsychiatric Phenotypes

In this section, we aim to review some of the genes that have been identified through the comparative analysis of the different datasets and are relevant to neuropsychiatric disorders. Due to space limitations, we are unable to review all the genes but the ones selected here are those that are involved in the MAPK signaling pathway, neuronal development, and synaptic plasticity.

### Mapk10/JNK3

Mitogen-activated protein kinase 10 (*Mapk10*, or also known as c-Jun N-terminal kinase, *JNK3*), a known effector of Rho-GTPase kinase, is enriched in the nervous system (Mohit et al., 1995). Although the *Mapk10* gene was shown to associate with the *miR-128* binding complex in the Tan study, the expression of *Mapk10* is not upregulated in *miR-128*-deficient D1 neurons (Tan et al., 2013). Whilst there was no change in the D1 neurons, *MAPK10* is still an important gene to consider as a *miR-128* target and in a neuronal context due to its role in phosphorylating and binding PSD-95 in dendritic spines (Kunde et al., 2013). In addition, patients with chromosomal translocations at the *MAPK10* locus present with epileptic seizures, motor and cognitive delays (Shoichet et al., 2006; Kunde et al., 2013) and a recent behavioral study by Reinecke et al. (2013) reported that *Mapk10* knockout mice showed signs of anxiety as they were less active and did not exhibit normal navigational behavior during the Morris water maze task. The authors hypothesize the behavior could be due to impaired neuronal plasticity as MAPK/JNKs are involved in synaptic development (Tararuk et al., 2006).

### Arpp21/Rcs

*ARPP21*, a cytosolic neuronal phosphoprotein, is highly expressed in the mammalian central nervous system especially within the basal ganglia (Walaas et al., 1983; Ouimet et al., 1989). *Arpp21* functions as a calmodulin (CaM) signaling regulator (and it is also known as Regulator of calmodulin signaling, *Rcs*) (Rakhilin et al., 2004). CaM plays an important role at the synapse as it regulates the release of neurotransmitters from the presynaptic terminal (Ando et al., 2013). Phosphorylation of serine residue 55 (Ser55) by protein kinase A (PKA) in *Arpp21* also leads to an increased binding to CaM, which inhibits the CaM activity, and in turn affects calcineurin, a CaM-dependent phosphatase 2B, and CaM kinase I (CaMKI) activities (Williams et al., 1989; Rakhilin et al., 2004). PKA phosphorylates *Arpp21* at the Ser55 residue and this phosphorylation can be modulated by D1 and D2 receptor agonists whereby, there was increased phosphorylation upon D1 receptor agonist treatment, but decreased phosphorylation when exposed to D2 receptor agonists (Caporaso et al., 2000). In addition, this phosphorylation is sensitive to methamphetamine and cocaine (Caporaso et al., 2000), which is not surprising, given its expression within the limbic system. Taken together, data from several studies (Megraw et al., 2010; Davis et al., 2012; Tan et al., 2013) and its role in the CAM kinase pathway strongly indicates a potential role for *Arpp21* in regulating the development of neuropsychiatric symptoms.

There are six out of seven genes (nine genes in total excluding *Mapk19* and *Arpp21*; **Figure 1A**) present in Dataset TS, T, and P that have been reported to be associated with neurologically relevant functions (**Table 1**). For the seventh gene, cancer susceptibility candidate 3 (*Casc3*, or known as *Mln51*), most reports link this gene to non-sense-mediated mRNA decay (Chang et al., 2007) and the exon junction complex (Chazal et al., 2013) but there are no reports so far of any association with neurological/neuropsychiatric functions.

In addition to the candidate genes (which were shared by all datasets) reviewed above and in **Table 1**, we also reviewed other genes with highly suggestive neuropsychiatric associations, which were identified either through the TargetScan prediction and/or through literature reviews.

### Reln

Reln is an extracellular glycoprotein secreted by Cajal-Retzius cells during prenatal development (Del Rio et al., 1997) and has an important role in neuronal migration (Frotscher et al., 2009). *Reln* is a direct target of *miR-128* (Evangelisti et al., 2009; Lin et al., 2011).

*RELN* expression is downregulated in schizophrenia, bipolar disorders (Guidotti et al., 2000), autism (Fatemi et al., 2005), and Alzheimer’s disease (Herring et al., 2012). Polymorphisms in *RELN* associate with the risk of developing schizophrenia in the Han Chinese female population (Kuang et al., 2011), but this has not been corroborated by Ovadia and Shifman (2011). Rather, what they found was that the shorter isoform of *RELN* was significantly reduced in bipolar samples and an allelic imbalance of *RELN* expression in schizophrenia samples. The authors speculate that epigenetic factors or genetic imprinting may be responsible for the abnormal RELN function in these disorders.

*Reln* homozygous mutant mice have abnormal brain development and an enhanced rate of seizures (Patrylo et al., 2006; Qiu et al., 2006). In addition, Qiu et al. (2006) observed a reduction in freezing behavior (animal stops moving due to fear) which is associated with a defect in associative learning but other anxiety-like behaviors were normal. Similarly, no stress- and anxiety-like behaviors were found in *Reln* heterozygous mice (Teixeira et al., 2011), but they found that mice with *Reln* overexpressed were less susceptible to depressive-like behaviors and were resistant to chronic cocaine stimulation.

An interesting study has shown that cleavage at a particular site on the RELN protein (known as N-t), can sustain RELN activity beyond the normal range of the wildtype protein in an *in vitro* model (Koie et al., 2014), which may point to a potential therapeutic target for certain neuropsychiatric disorders.

### Dcx

DCX is a microtubule associated protein which binds to microtubules to enable neuronal migration (Koizumi et al., 2006; Moores et al., 2006). DCX is also involved in the dynamic development of dendrites through regulating their length, branching point and complexity of the dendrites (Cohen et al., 2008). In addition, DCX regulates the distribution of neurofascin, a cell surface adhesion molecule at the outer surface of the cell in a microtubule-independent manner (Yap et al., 2012), which in turn, regulate axonal outgrowth and the formation of GABAergic synapses. *DCX* mutations are commonly associated with lissencephaly, smooth brain disorder, a neuronal migration disorder which then leads to epilepsy and mental retardation (Jang et al., 2013).

*MiR-128* inhibits the expression of *Dcx* by directly binding on its 3′UTR region and inhibition of *miR-128* successfully restores the expression of *Dcx* (Evangelisti et al., 2009). *Dcx* mutant mice exhibit a range of abnormal behaviors such as hyperactivity, spontaneous seizures, impaired social interaction, and decreased aggression (Nosten-Bertrand et al., 2008; Germain et al., 2013). Disorganized hippocampal CA3 regions have been observed (Nosten-Bertrand et al., 2008) and may account for the various behaviors, but a recent study indicated that the typical hippocampal-dependent behaviors such as spatial memory were not impaired in *Dcx* knockout mice (Germain et al., 2013).

## Conclusion

In this article, we sought to evaluate the potential role of *miR-128* target genes in neuropsychiatric disorders. We noticed that out of the 801 genes predicted to be targets of *miR-128* by TargetScan, only a small number of genes have been empirically proven to be regulated by this miRNA. This indicates a large void of knowledge of which genes are true targets of *miR-128*, and more research is needed to construct a broader picture of the pathways regulated by *miR-128.*

Based on a systematic review and analysis of predicted and current empirically determined targets of *miR-128*, we found that *miR-128* regulates many pathways within the cell, including pathways involved in cancer or neuronal maturation. With respect to neuropsychiatric phenotypes, we found an association with many *miR-128* target genes. As many of these have well characterized functions, we postulate that therapeutic targets can be directed toward these known pathways and the central role *miR-128* plays in these pathways may indicate a potential role as a biomarker for selected neuropsychiatric disorders.

## Conflict of Interest Statement

The authors declare that the research was conducted in the absence of any commercial or financial relationships that could be construed as a potential conflict of interest.
