# Supplementary material for: A Perspective on the Role of microRNA-128 Regulation in Mental and Behavioral Disorders
Source: Front Cell Neurosci. 2015 Dec 14;9:465. doi: 10.3389/fncel.2015.00465 (PMC4677093; doi:10.3389/fncel.2015.00465)
Supplement: Supplementary file 6 [file Image_1.PDF]

Supplementary Material

A perspective on the role of *microRNA-128* regulation in mental and behavioral disorders

Ai-Sze Ching[1], Azlina Ahmad-Annuar[1]*

[1]Department of Biomedical Science, Faculty of Medicine, University of Malaya, Kuala Lumpur, KL, Malaysia.

***Correspondence:** Azlina Ahmad Annuar**,** Department of Biomedical Science, Faculty of Medicine, University of Malaya, 50603 Kuala Lumpur, Malaysia.

azlina_aa@um.edu.my

1. Supplementary figure

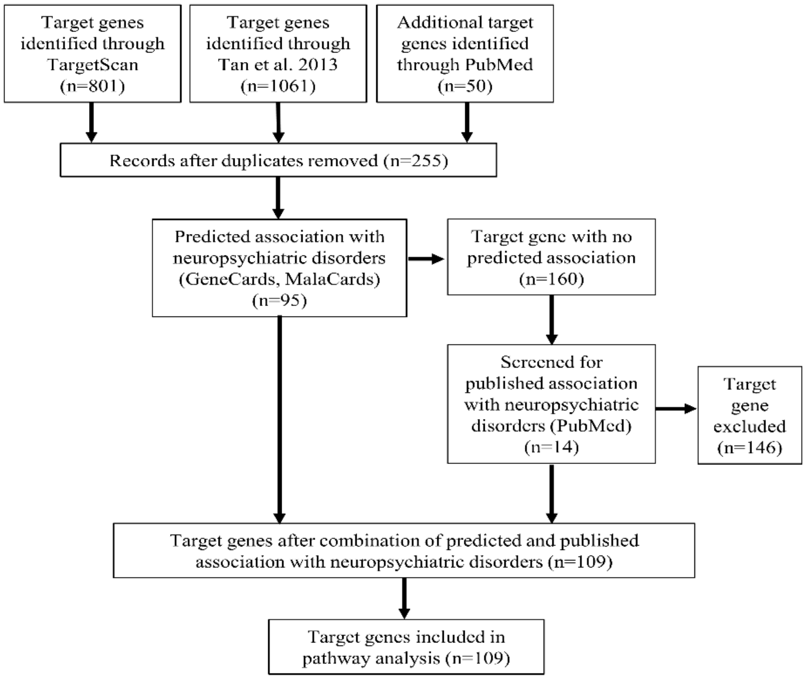

Supplementary Figure 1. The analytical workflow of Datasets TS & T & P to select genes with an association with neuropsychiatric phenotypes. 255 genes were selected and 95 genes were predicted to be associated with neuropsychiatric disorders by GeneCards, the rest of the genes were subjected to systemic review on PubMed. 14 genes were reported to associate with neuropsychiatric disorders. A total of 109 genes (Data NP) were found to be associated with neuropsychiatric disorders and were subjected to DAVID bioinformatics analysis.
